# Evaluating organizational change in health care: the patient-centered hospital model

**DOI:** 10.1186/s12913-018-2877-4

**Published:** 2018-02-08

**Authors:** Carlo V. Fiorio, Mara Gorli, Stefano Verzillo

**Affiliations:** 1 European Commission, Joint Research Centre**, Via E. Fermi, 2749, Ispra (VA), 21027 Italy; 2 Irvapp-FBK, Via Santa Croce 77, Trento, 38122 Italy; 3 CRISP - Interuniversity Research Centre on Public Services, Universitá degli Studi di Milano-Bicocca, Piazza dell’Ateneo Nuovo, 1, Milano, 20126 Italy; 4 Universitá Cattolica del Sacro Cuore, Largo Gemelli, 1, Milano, 20123 Italy; 5 Universitá degli Studi di Milano, Via Conservatorio, 7, Milano, 20121 Italy; 6 Dondena Centre, Bocconi University, Via Rontgen, 1, Milano, 20136 Italy; 7 CERISMAS, Centro di Ricerche e Studi in Management Sanitario c/o Universitá Cattolica del Sacro Cuore, Via Necchi 7, Milano, 20123 Italy

**Keywords:** Patient centered model, Hospital change, Ex-post evaluation, Difference-in-difference, Efficiency, Effectiveness, Administrative data, Major diagnostic categories, Hospital discharge charts, Italy

## Abstract

**Background:**

An increasing number of hospitals react to recent demographic, epidemiological and managerial challenges moving from a traditional organizational model to a Patient-Centered (PC) hospital model. Although the theoretical managerial literature on the PC hospital model is vast, quantitative evaluations of the performance of hospitals that moved from the traditional to the PC organizational structure is scarce. However, quantitative analysis of effects of managerial changes is important and can provide additional argument in support of innovation.

**Methods:**

We take advantage of a quasi-experimental setting and of a unique administrative data set on the population of hospital discharge charts (HDCs) over a period of 9 years of Lombardy, the richest and one of the most populated region of Italy. During this period three important hospitals switched to the PC model in 2010, whereas all the others remained with the functional organizational model. This allowed us to develop a difference-in-difference analysis of some selected measures of efficiency and effectiveness for PC hospitals focusing on the “between-variability” of the 25 major diagnostic categories (MDCs) in each hospital and estimating a difference-in-difference model.

**Results:**

We contribute to the literature that addresses the evaluation of healthcare and hospital change by providing a quantitative estimation of efficiency and effectiveness changes following to the implementation of the PC hospital model. Results show that both efficiency and effectiveness have significantly increased in the average MDC of PC hospitals, thus confirming the need for policy makers to invest in new organizational models close to the principles of PC hospital structures.

**Conclusions:**

Although an organizational change towards the PC model can be a costly process, implying a rebalancing of responsibilities and power among hospital personnel (e.g. medical and nursing staff), our results suggest that changing towards a PC model can be worthwhile in terms of both efficacy and efficiency. This evidence can be used to inform and sustain hospital managers and policy makers in their hospital design efforts and to communicate the innovation advantages within the hospital organizations, among the personnel and in the public debate.

## Background

In recent decades, national health care systems have been dealing with an increased demand for high-quality and patient-centered services, but limited resources have often challenged their sustainability ([1]). New demands and needs are emerging, connected with the growth of chronic pathologies, the ageing of the population, the development of technologies, the scarcity of economic resources and people’s emerging awareness of their care and cure rights. With respect to this demographic, epidemiological and social context, health care and hospital systems overall must innovate to respond to the new care needs. The mandate to “do more with less” encourages policy makers, health care managers and scholars to look for innovative ways to redesign health care services. The need for innovation is often interlaced with processes of organizational redesign in many forms. There are many examples of health care organizations that have committed to broad changes due to the actual social and economic demands. A significant stream of change relates to technological innovations, such as telemedicine ([2]). There exists extensive experience of activation of new social and integrated care networks. These are designed to act as community-based care networks ([3, 4]). A major movement in policy making identifies the “patient-centered approach” as the key leverage for making the health care delivery system respectful of, and responsive to, the current needs and requirements ([5–8]). The patient-centered approach, while presenting clear statements, principles of care and operative practices, also leads to different care model designs within hospitals ([9]). In fact, an increasing literature ([10–13]) suggests that innovation in health care should evolve towards a patient-centered (henceforth PC) model, reshaping hospitals with the aim of moving from functional towards process-oriented organizational forms, focusing on the process of care instead of on functional, self-referential departments within the hospital. To innovate towards the PC model, hospitals usually undergo a process of redesign that encompasses several restructuring actions, both in the organizational structure and in the physical building ([14]).

Although the theoretical managerial literature on the PC model is vast, evaluations of the performance of hospitals that have moved from the functional to the PC organizational structure are scarce (with a few exceptions, such as [11, 15, 16]). The complexity of the variables at play, the sensitivity of data, which are not always made available for research, the diversity of the pathologies and types of patients and many other elements have so far made the construction of a methodological framework for the evaluation of the PC hospital model extremely challenging. The shift to different hospital models may therefore follow international trends and interests that not always are connect to clear *ex ante* impact evaluation ([17]). However, without any evaluative research, any innovation risks being perceived by local communities and by organizations’ employees as being driven more by political reasons or managerial trends than by a serious assessment of its benefits in terms of effectiveness and efficiency. In this work, we take the challenge to embark on a sound assessment of the efficiency and effectiveness of the PC model as opposed to the traditional functional-based hospital model. To approach the PC model evaluation, we begin by considering and evaluating two assertions that constitute the essential policy makers’ drivers for innovating towards the PC model:

- the PC model responds to the need to reduce waste, hence increasing hospital efficiency;
- the PC model responds to the need to reshape care delivery processes around the needs of the patients, increasing the effectiveness of the treatment ([12, 18]);

Driven by the belief that an assessment of important organizational changes is crucial, we show how this is possible given the availability of a quasi-experiment and of adequate administrative data. Our research study focuses on the provision of health care services in the Lombardy region, the richest and one of the largest regions of Italy. With nearly 10 million inhabitants, Lombardy is larger than the median country in the EU by population and one of the richest region of Europe by per capita GDP. In this context, three important hospitals switched to the PC hospital model at the end of 2010, while the rest of the Lombardy hospitals remained with the traditional functional organizational structure. In this paper, we suggest an empirical strategy for a quantitative evaluation of the overall impact of the PC model on the pre-existing one, following traditional evaluation studies, in which the effects of a policy intervention are measured through appropriate econometric techniques (difference in difference estimators) on a set of selected outcome indicators (e.g. [19]). The available data for this research, based on an administrative data set, are used to measure the effectiveness and efficiency by major diagnostic category (henceforth, MDC). The relevance of this study is related not solely to evaluate the PC hospital model impact, which is proposed as the main focus of our analysis. Our research exercise suggests that ex-post assessment of organizational changes by the use of statistical data is relevant for informing about policy implications and serve as a driver for future innovations.

### The patient-centered hospital model

Hospitals have often been conceived as functional organizational structures, in which patients requiring a similar area of expertise are grouped into independently controlled departments. Although in some countries such organization seemed for a long time to be the most appropriate to support and foster the knowledge development required by medical science, the functional structure has shown severe shortcomings, consisting mainly of economic and organizational inefficiencies. In fact, the functional organization often lacks the capability to control the work flow across departments and thus the coordination of the care activities within a patient care trajectory. Moreover, in the functional organization, resources tend to be duplicated, causing waste, and the autonomy in using the specialty’s resources often prevails over accountability, in some cases reducing the effectiveness of treatments ([10, 12, 20]). The inefficiencies and complexities detected in functional hospital organization led to many forms of organizational innovation. Examples may be found in the process-oriented design ([11, 20]), in the lean philosophy ([21]) or in the experimentation of new hospital settings ([9]). Another planned change process is the one defined as the patient-centered (PC) hospital model, towards which hospitals are converging worldwide, for instance in England ([22]), the Netherlands ([23]), Spain ([24]), Sweden ([25]) and Italy ([26]). The PC model represents an attempt to redesign the care delivery process by shaping the structures and processes involved in delivering hospital care according to the needs of the patients. In the traditional hospital models, patients are admitted under individual specialist clinicians, who keep them or transfer them to the care of another clinician.

As summarized in Table 1, to innovate toward the PC model, hospitals undergo a process of redesign that encompasses several restructuring actions that, by taking stock from authors (cfr. [10, 20, 27]) we summarize over six dimensions ([28]). The first regards the change of the organizational model, which passes from a functional/divisional model to a process-oriented model ([20]). The second is the transformation of the concept of organizational unit, necessary for responding to patients’ care needs and for managing the relationship among specialties. The criteria for patients’ allocation to hospital units switch from specialty-based units to multi-specialty units, differentiated by the level of patients’ clinical and assistential care needs instead of by their specific pathologies. In fact, the core principle of the PC model consists of the delivery of the appropriate amount of cure and care to patients in the most suitable setting according to their health conditions. Third, as the PC model requires integrated care, multi-professional and multi-specialty teams are strengthened and requested to collaborate. This is consistent with a different analysis proposed for patient centeredness carried out by [29] and by [30]. An example of this new integrated effort is represented by the specific reconfiguration of nurses’ position, in which the traditional “functional nursing” (i.e. nurses specializing in a single care activity) becomes “modular nursing” (i.e. nurses responsible for the overall assistential practices required by small groups of patients within the ward). Fourth, hospitals rethink their use of resources, such as beds, operating rooms and equipment, which are shared by all the functional specialties and they, regroup and regulate them by a centralized logistical model. Patients are no longer transferred across different units or departments; rather, physicians and technologies move to the patients’ bed. Fifth, such re-organization calls for new managerial roles ([10]) responsible for the appropriateness, timeliness, flow and integration of patients’ care delivery process (e.g. the bed manager or case manager). Sixth, the described changes might require a redesign of the physical environment to maximize the resource pooling and the patients’ grouping based on the patients’ clinical severity and on the complexity of the assistance required ([27]).

**Table 1:** Disentangling the differences between traditional and PC hospitals

	Functional hospital configuration	More recent innovations: converging patterns towards PC hospitals
Organizational model/ care delivery model	Functional/divisional model	Lean organization/process-oriented model
Organizational unit: patients’ care needs and the relationship among specialties	Specialty-based units. Practitioners (doctors and nurses) are grouped into semi-autonomous units depending on their specialty of belonging	Multi-specialty units. Units are aggregated in accordance with patients’ clinical and assistential needs. Doctors might treat patients located in different units and nurses might assist patients with different pathologies
Model of care	Functional nursing (nurses’ task-oriented job: each nurse is specialized in a single care activity)	Modular nursing (nurses are responsible for the overall assistential practices required by small groups of patients within the ward)
Use of resources	Separated resources (beds, operating rooms, equipment, nursing staff, other staff) devoted to the individual specialties	Resource pooling: resources are shared by all the functional specialties regrouped
Managerial roles	Head physicians in charge of their departments	Bed manager/case manager (as distinguished by the clinical activity) for centralized operation management
Physical environment	Hospitals are built around fixed and focused spaces, with often isolated wings	Newly built hospitals are designed to maximize resource pooling and patient grouping, flexibility and modularity of spaces

The PC organizational model is understandably characterized by local variations depending on the boards’ strategic choices, the hospitals’ dimensions, the workforce composition, the patients’ average characteristics, and so on. While this type of diversity is hardly predictable and should be better addressed by case study analyses ([31, 32]), the main common traits of the PC innovation can be identified, provided that a suitable environment and adequate data are available. For the former, one needs a context in which, from a pool of comparable units before treatment, some hospitals have been treated while others have not. For the latter, one needs data characterized by minimal error due to mis-measurement, a non-random response rate and proper population coverage. Unsurprisingly, there are very few studies providing ex post analysis of the implementation of the PC model so far. The application of the PC principles is expected to improve quality, increase patient satisfaction, increase job satisfaction for staff and improve efficiency ([33]). Reports on new PC - hospitals highlight the positive aspects of patient-friendly and staff-friendly design ([34]). Other authors, however, question the strength of these claims ([18, 22]). A few authors (see for example [10, 20]) present extensive literature reviews on assessing hospitals’ changes and hospital designs (see for example [35]), thus ending up tracing the factors that affect their success or failure in the redesign process but provide no ex post analysis of the PC model adoption. To the best of our knowledge, there is still little evidence either to support or to refute these claims, notably in the European context ([36]), and there is no quantitative assessment of the efficiency and effectiveness of the PC model as a whole. Considering the relevance of the PC model change with respect to hospital managing and policy making, and considering also the extensive implementation and debate in European countries and international context, this paper proposes to fill the quantitative assessment gap, with a specific focus on efficiency and effectiveness of PC implementation.

## Methods

### The empirical model

A key ingredient in assessing the effects of a change from a functional to a PC model is to observe, in a group of comparable hospitals, a change in a group of hospitals (treated units) as opposed to others (control units) over time. The decision to move from a functionally organized to a PC hospital model is typically taken at the hospital level; however, its implementation might differ greatly depending on each major diagnostic category^1^, as some MDCs are more influenced by the organization, whereas others follow very strict protocols regardless of the organizational model adopted. In our model, we identify the effect of moving from a functional to a PC model of hospital organization, exploiting the variability of health outcomes across MDCs. For such an organizational change, there is no need for high-frequency data (e.g. daily), as it is likely to have an impact on the hospital performance over months or years, or for individual data, as the focus is on the average efficiency and effectiveness in MDCs of treated hospital units versus those in untreated ones. However, such an empirical setting requires the availability of large data sets regarding the characteristics of all the MDCs in several hospitals over time. The increasing availability of administrative data about hospital discharge charts (henceforth, HDCs) allows us to overcome this major data requirement.

As we have access to administrative data on the full population of all HDCs for all Lombardy hospitals between 2004 and 2012, we managed to build some measures of effectiveness and efficiency by MDC. In our empirical model, we organize the data by year of discharge and collapse the data by the average HDC at MDC *j* in hospital *h* at time *t*. The reason for keeping the MDC dimension in our collapsed data is that hospitals differ greatly in terms of the MDC mix and relative importance and we aim to exploit this variability for the identification of our main coefficient as well. The basic model is a standard difference-in-difference model:

1 $$ \begin{aligned} y_{j,h,t}&=Z_{j,h}+T_{t}+\alpha_{1}{HDC}_{j,h,t}+\alpha_{2}{Age}_{j,h,t}\\ &\quad+\alpha_{3}{Male}_{j,h,t}+\gamma {PC}_{h,t}+\epsilon_{j,h,t} \end{aligned}  $$

where *y*_*j*,*h*,*t*_ is the logarithmic transformation of the average outcome^2^ in MDC *j* of hospital *h* at time *t*, *Z*_*j*,*h*_ are fixed effects identifying idiosyncratic characteristics of MDC *j* in hospital *h* and *T*_*t*_ are year fixed effects that account for possible common trends, such as technological advancement or a changed demand for certain services. We also control for a set of variables defined at the *j*,*h*,*t* cell level, such as the average number of discharges (*HDC*_*j*,*h*,*t*_), the average age of patients (*Age*_*j*,*h*,*t*_) and the share of male patients (*Male*_*j*,*h*,*t*_). The variable *PC*_*h*,*t*_ is defined as a dummy that is equal to one if the PC has been adopted in hospital *h* in year *t* and zero otherwise^3^, and *ε*_*j*,*h*,*t*_ is an error term. By controlling for a set of observables over time, we control for observed differences among the treated and the control group, which allows us to reduce the imbalance of the two samples. The main coefficient of interest is *γ*, which accounts for the difference in the logarithm of the mean outcome due to the adoption of the PC organizational method. However, the estimate of *γ* could be biased by a set of omitted variables, which could take into account the fact that hospitals’ heterogeneity depends on the know-how developed in each MDC, which typically increases with the number of patients treated, on the morbidity of the average patients in each MDC and on their age and gender. The heterogeneity of MDCs within hospitals also affects the heterogeneity among hospitals that a simple hospital fixed effect, such as the one used in the basic specification (Eq. 1), would be unable to capture.

Hence, we also control for a set of interaction terms, which are introduced into the basic model incrementally to reach a saturated one. In particular, we first condition on the interaction of year fixed effects with MDC dummies (*I*_*j*_×*T*_*t*_, where *I*_*j*_ is equal to 1 for MDC *j* and 0 otherwise) and with hospital dummies (*I*_*h*_×*T*_*t*_, where *I*_*h*_ is equal to 1 for hospital *h* and 0 otherwise) to account for possibly different time trends among different MDCs and hospitals. We then control for the interactions of the average number of discharges with MDC dummies (*I*_*j*_×*HDC*_*j*,*h*,*t*_) and with hospital dummies (*I*_*h*_×*HDC*_*j*,*h*,*t*_) to account for heterogeneity in the attractiveness of hospitals and the frequency of diagnostic categories. Finally, to take into account patient complexity and risk adjustment issues, we also control for the interactions of the average age of patients with MDC dummies (*I*_*j*_×*Age*_*j*,*h*,*t*_) and with hospital dummies (*I*_*h*_×*Age*_*j*,*h*,*t*_) to account for heterogeneity in the age composition of discharges by MDCs and hospitals and for the interactions of the share of male patients with MDCs (*I*_*j*_×*Male*_*j*,*h*,*t*_) and hospitals (*I*_*h*_×*Male*_*j*,*h*,*t*_), since different diagnostic categories are characterized by different gender compositions of patients. The saturated model that we finally estimate can be written as follows:

2 $$ \begin{aligned} {}y_{j,h,t}=&Z_{j,h}+T_{t}+\alpha_{1}{HDC}_{j,h,t}+\alpha_{2}{Age}_{j,h,t}+\alpha_{3}{Male}_{j,h,t}\\ &+\beta_{1}I_{j}\times T_{t}+\beta_{2}I_{h}\times T_{t}\\ &+_{3}I_{j}\times {HDC}_{j,h,t}+\beta_{4}I_{h}\times {HDC}_{j,h,t}\\ &+\beta_{5}I_{j}\times {Age}_{j,h,t}+\beta_{6}I_{h}\times {Age}_{j,h,t}\\ &+\beta_{7}I_{j}\times {Male}_{j,h,t}+\beta_{8}I_{h}\times {Male}_{j,h,t}\\ &+\gamma {PC}_{h,t}+\epsilon_{j,h,t} \end{aligned}  $$

By including all the possible pairwise interactions, we identify the coefficient of interest by estimating the empirical models outlined above by ordinary least squares, assuming that the remaining variation is explained by the dummy variable, which identifies the adoption of the PC model. From a methodological point of view, over-controlling in a linear regression model is similar to statistical matching (e.g. propensity scoring) and the models deliver very similar results (among others, see [37]). To account for the presence of a common random effect at the hospital level, all the models are estimated with clustered standard errors at the hospital level.

### Data and performance measures

We use a large administrative data set covering the full population of patients and hospitals operating in the Lombardy Health Care System. Our data set combines information on more than 17.4 million hospital discharge charts (HDCs), over 25 MDCs, provided by all Lombardy hospitals, concerning 13.3 million patients between 2004 and 2012^4^. They are individual records with daily frequency, but since we focus here on the average efficiency and effectiveness of MDCs in hospitals that moved to a PC organization as compared with those in hospitals that maintained the traditional organization, we consider the yearly frequency of the average HDC.

The administrative data set that we use is routinely collected by hospitals for both financial and managerial purposes and is relayed regularly to the regional administration. The main advantages of using administrative records consist of full population coverage and the significant reduction of measurement and sampling errors, with plenty of details about the diagnosis and the service provided. Each HDC reports information regarding the patient characteristics (gender, age and province of residence) and the discharge characteristics (e.g. diagnosis-related group^5^, length of stay in hospital, major diagnostic category, regional reimbursement, number of times the patient was physically or administratively transferred within the same hospital before discharge^6^, etc.). This data set has been linked with other information, also provided by the Lombardy Health Care Department, regarding several hospital characteristics, such as ownership and geographic location. These data are also matched with the registry office that records the deaths of all residents in the region.

According to the international literature ([38, 39]), outcome indicators of hospital care essentially analyze costs in relation to some proxies for the quantity of delivered care. Although these outcomes are not entirely under the control of the hospitals, they deal with the risk of adverse events (effectiveness) as well as with the hospitals’ ability to satisfy the care demand (efficiency) ([40]). Moreover, outcomes indicators have high relevance from the viewpoint of both patients and policy makers as reliable proxies for health care quality^7^.

Our data set allows us to define a limited number of efficiency and effectiveness outcomes. Here, as a measure of efficiency, we consider the following index:

1. *Average days of stay in hospital*: this index counts the average number of days from admission to the hospital to discharge.

It provides a measure of efficiency as, by reducing the length of stay (LoS) a hospital would manage to reduce its costs. As for the effectiveness measures, we consider the rate at which patients are re-hospitalized in the same major diagnostic category (MDC) within 30 days (both in the same hospital and in different hospitals), as *ceteris paribus* this might signal an early discharge or unsatisfactory treatment. Related to this, we would also like to test whether patients treated in PC hospitals have different mortality rates from those treated in traditionally organized ones. The literature studying acute care typically focuses on in-hospital mortality, possibly also because of the difficulty of reporting accurately all discharged patients’ deaths. In fact, our administrative data record whether any discharged patients died at any moment after the day of discharge up to the end of 2012, allowing us to construct a mortality rate within 30 days of discharge, which is likely to provide an accurate indication of care effectiveness ([41]). We have no a priori expectation regarding how the PC organization could affect this outcome variable. It might even be that for such an important health care outcome, the PC innovation will be found to have no significant effect. Hence, we consider three effectiveness indexes based on the available information:

1. *Average number of readmissions within 30 days*: this index measures the number of readmissions of the same patient to a Lombardy hospital within the same MDC within 30 days of discharge;
2. *Average number of readmissions in the same hospital within 30 days*: this index measures the number of readmissions of the same patient to the same hospital and to the same MDC within 30 days of discharge;
3. *Average mortality rates within 30 days*: this index defines the mortality rate of patients within 30 days of hospital discharge.

In fact, this set of indexes provides only a partial picture of efficiency and effectiveness at the hospital level. For instance, one would like to measure efficiency also comparing costs and benefits of treatment, assess incentives provided to medical doctors and nurses, and measure effectiveness also analysing patients’ satisfaction and care quality, however our data do not provide such information and for their administrative nature they cannot be merged with other data sets.

### Sample selection and descriptive statistics

Before using the data set to estimate the empirical models outlined above, we discarded the discharge charts belonging to patients with a province of residence outside Lombardy, discharges for hospitalizations shorter than one day and subacute hospital discharge charts^8^. As all three hospitals that introduced the PC in the last quarter of 2010 (the Ospedale Civile di Vimercate, the Ospedale S. Anna di Como and the Ospedale di Legnano) are public and non-research-oriented hospitals, we selected only hospitals belonging to the same category. We also dropped a few other hospitals that could not be clearly ascribed to either the treated or the control group as some had started the PC model implementation before and some immediately after our observation period and those for which it was not possible to identify a clear starting point for the move to the PC model. As all the PC hospitals considered provide care to patients of any MDC, we also dropped those hospitals that did not present HDCs for all MDCs. Hence, we collapsed the data set by major diagnostic categories (MDCs)^9^, hospitals and year and dropped all the cells produced by the collapse with fewer than 30 discharges to preserve an acceptable level of precision^10^. Eventually, we obtained a panel of 25 MDCs belonging to 86 hospitals over at most 9 years (from 2004 to 2012), with a total size of nearly 13 thousand observations.

Table 2 shows some summary statistics of the total sample, showing that in the average MDC the average age is 51.77, 47.89*%* of patients are male and the number of discharges is about 522 per year. Table 3 shows some descriptive statistics of the efficiency and effectiveness outcomes for the PC and functional hospitals before and after the organizational change that took place at the end of 2010. The average number of days in hospital of average MDCs increased by 0.3 in PC hospitals as opposed to 0.41 in functional ones. The rate of re-hospitalization in the same hospital and in the same MDC decreased for all Lombardy hospitals after 2010 compared with the previous period, suggesting an overall increase in effectiveness, but the decrease was slightly larger in PC hospitals (− 0.008) than in functional hospitals (− 0.003). As we observe the full population of Lombardy hospitals, we can also observe the case of patients who needed re-hospitalization for the same MDC but decided to change hospital, possibly because they did not appreciate the treatment received in the first one. The descriptive statistics suggest that re-hospitalization for the same MDC but in different hospitals is slightly negative for the average MDC of PC hospitals (− 0.007) and slightly positive for the group of controls (0.002). As our administrative data are matched with registry office data recording people who passed away, we can also make a clear estimate of the mortality rate of patients after being discharged by a hospital. The average mortality rate for the average MDC is about 6% for PC hospitals and slightly higher for functional ones; however, what matters most for our research focus is that the change between before and after 2010 is very similar for both groups of PC and functional organization hospitals. The differences in the changes between pre- and post-treatment periods of average MDCs in the control and treated groups for the considered measures of efficiency and effectiveness suggest that some improvement might have been produced by the switch to the PC organizational model, but for a proper statistical assessment of their significance we need the estimation of the empirical model outlined above.

**Table 2:** Summary statistics of patients’ characteristics in average MDCs

Variable	Obs.	Mean	Std Dev.	Min.	Max.
*All hospitals*					
Share of male patients (%)	12,120	47.898	0.191	0	100
Average age	12,120	51.773	19.312	0	84.750
Number of HDCs	12,120	522.294	666.592	31	10,81

*Source*: Our calculations using data provided by the Lombardy Health Care Department

*Notes*: MDC averages out of 3 PC and 83 functional hospitals

**Table 3:** Summary statistics before and after the organizational change, in average MDCs

		LoS	Re-Admiss.1	Re-Admiss.2	Mortality
**PC hospitals**					
	Obs.	Mean.	Mean.	Mean.	Mean.
Before	441	7.210	0.045	0.058	0.050
After	126	7.509	0.037	0.051	0.063
*Change*		*0.300*	*-0.008*	*-0.007*	*0.012*
**Functional hospitals**					
Before	9,023	7.859	0.041	0.075	0.060
After	2,53	8.275	0.038	0.078	0.071
*Change*		*0.416*	*-0.003*	*0.002*	*0.011*

*Source*: Our calculations using data provided by the Lombardy Health Care Department

*Notes*: MDC averages out of 3 PC and 83 functional hospitals

Re-Admiss.1: Re-hospitalization rate (same hosp. & MDC) and

Re-Admiss.2: Re-hospitalization rate (MDC)

## Results

**Fig. 1:**
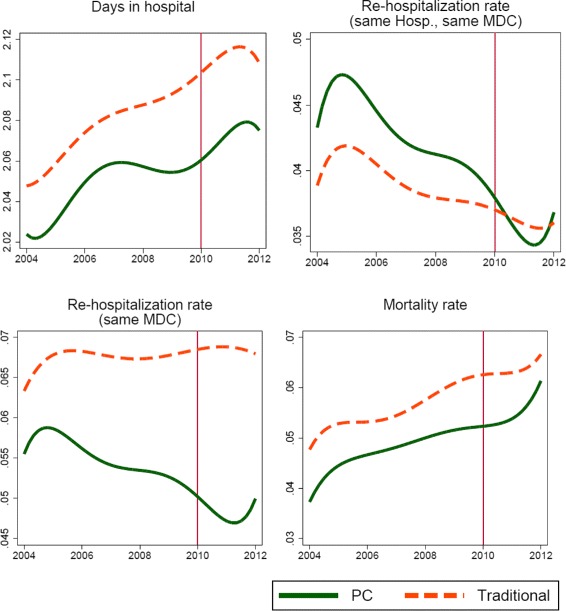
Parallel Trend

At the core of our difference-in-difference identification strategy lies the so-called parallel trends assumption. A graphical representation of the parallel trend assumption is provided in Fig. 1. However, as in some cases the graphical representation is not conclusive, we also tested the internal validity of our identification strategy by checking whether there is any evidence rejecting the assumption of parallel trends for the period before the treatment of PC and traditionally organized hospitals. The results are presented in Table 4, showing that there is no evidence to reject the parallel trends assumption^11^, hence we proceed presenting our main results.

**Table 4:** Test for parallel trends of treated and control hospitals in the period before the PC organizational change

Variable	(A)	(B)	(C)	(D)
Days in hospital				
F-stat.	0.026	0.007	0.001	0.001
Re-hospitalization rate (same MDC)				
F-stat.	0.059	0.002	0.004	0.002
Re-hospitalization rate (same Hosp., same MDC)				
F-stat.	0.068	0.003	0.013	0.012
Mortality rate				
F-stat.	0.095	0.008	0.001	0.003
MDC x Year interaction	No	Yes	Yes	Yes
Hosp. and MDC Interactions with:				
Discharges	No	Yes	Yes	Yes
Age	No	No	Yes	Yes
Male	No	No	No	Yes

Source: Our calculations using data provided by the Lombardy Health Care Department. *Note:* The table shows the test of parallel trends, for different outcome variables, respectively the log of the number of days of hospitalization (outcome days in hospital), the log of the rate at which a discharged patient happens to be re-hospitalized in the same MDC and in both the same hospital and MDC within 30 days (outcome re-hospitalization rates), the log of the mortality rate within 30 days after discharge (outcome mortality rate). The test is run for the period 2004–2010, fitting a fourth-order polynomial of a time trend

Table 5 shows our main results. This table presents the estimate of the *γ* coefficients; the empirical model is as outlined in the Material and Methods section and the whole list of efficiency and effectiveness measures is as above. Each coefficient estimate comes from different regressions, in which only the estimate of our coefficient of interest, its standard error in brackets and the total number of observations are presented. This offers us an immediate analysis of the overall effect on the average MDC of adopting PC organization in health care in the outcome analyzed.

**Table 5:** The effect of the PC organizational change, difference-in-difference estimations

	(1)	(2)	(3)	(4)
Days in hospital	-0.006	-0.033***	-0.042***	-0.046***
	[0.020]	[0.008]	[0.009]	[0.010]
No.Obs.	12,120	12,120	12,120	12,120
R-squared	0.943	0.962	0.965	0.966
Re-hospitalization rate (same MDC)	-0.007***	0.004*	-0.006***	-0.004*
	[0.002]	[0.002]	[0.002]	[0.002]
No.Obs.	12,120	12,120	12,120	12,120
R-squared	0.943	0.962	0.965	0.966
Re-hospitalization rate (same Hosp., same MDC)	-0.004	0.000	-0.006***	-0.006***
	[0.002]	[0.001]	[0.002]	[0.002]
No.Obs.	12,120	12,120	12,120	12,120
R-squared	0.871	0.899	0.904	0.909
Mortality rate	-0.000	-0.002	-0.000	0.001
	[0.002]	[0.002]	[0.002]	[0.002]
No.Obs.	12,120	12,120	12,120	12,120
R-squared	0.882	0.915	0.924	0.927
MDC x Year interaction	No	Yes	Yes	Yes
Hosp. and MDC Interactions with:				
Discharges	No	No	Yes	Yes
Age	No	No	No	Yes
Male	No	No	No	No

*Note:* The table shows the estimate of the coefficient of the PC variable, for different variables, and respectively the log of the number of days of hospitalization (days in hospital), the log of the rate at which a discharged patient happens to be re-hospitalized in the same MDC within 30 days (re-hospitalization rate, same MDC), the log of the rate at which a discharged patient happens to be re-hospitalized in the same hospital and MDC within 30 days (re-hospitalization rate, same Hosp. and same MDC) and the log of the mortality rate within 30 days after discharge (mortality rate). All regressions are estimated always controlling for the year fixed effects, average number of discharged patients, average age and share of male patients for MDC *j*, hospital *h* and year *t*. Additional interaction terms have been added sequentially, as shown at the bottom of the table. Standard errors have been clustered at the hospital level. All MDCs out of 3 PC and 83 functional hospitals

* 10% significance level, ** 5%, *** 1%

Column (1) presents the results for the basic model (Eq. 1), always including the year fixed effects, average number of discharged patients, average age and share of male patients by MDC *j*, hospital *h* and year *t*. In column (2) we add the interactions between hospitals and MDC fixed effects and the number of discharges to capture effects that could be hospital-specific, MDC-specific or size-specific. We also add the interactions between hospital and MDC dummies in column (3) with the average age and in column (4) with the average gender composition of each cell, to capture the compositional differences of MDC’ hospital cells. The estimate of *γ* for the saturated model of Eq. 2 is then presented in column (4). All the models are estimated with cluster-corrected standard errors at the hospital level.

Column (1) of Table 5 shows that, on the one hand, there is no evidence that PC hospitals deal with higher levels of efficiency (their coefficient are not statistically different from zero), while, on the other hand, we find significant evidence of higher levels of effectiveness of PC hospitals in terms of the re-hospitalization rate in the same MDC. However, once we control for the interaction of MDCs, year dummies and number of discharges in each cell (column 2) and eventually reach the fully saturated model (column 4), all the coefficients become statistically significant, suggesting that, taking into account the average heterogeneity among MDCs, the PC organizational model has an effect on both the selected efficiency and the selected effectiveness outcomes.

These results suggest the following conclusions. The PC organizational model significantly increases hospitals’ efficiency, reducing the length of hospitalization (− 4.6*%*). This estimate rises strongly when heterogeneity in the number of discharges by MDC is taken into consideration in addition to the year-specific interactions, as the *γ* coefficient estimate jumps from about − 0.015 to − 0.069 from column (1) to column (4).

However, in addition to the predictable higher level of efficiency associated with the PC model, one should also expect an impact in terms of effectiveness, looking at the average re-hospitalization rate within 30 days of discharge for the same MDC and for the same MDC and hospital and on the mortality rate at 30 days. We find no statistically significant reduction of the mortality rate (the estimated coefficient is 0) but a relatively more important reduction in both the re-hospitalization rates of discharged patients. Column (4) suggests that, having controlled for average patients’ age and gender composition of the hospital and MDC, the rate of re-hospitalization reduces slightly but significantly, by 0.6*%* within the same MDC and hospital and by 0.4*%* within the same MDC only. This is a relevant drop, which immediately affects the welfare of discharged patients.

There are, however, some *caveats* that should be stressed. First, there is the role of possibly confounding factors, which could bias our estimates. For instance, the transition to a PC model from a traditional organizational model involves changing incentives, for medical doctors, for nurses and for managers, but to account for them we should have access to detailed information about the composition of the hospital workforce and its remuneration and incentive policies. This is something that unfortunately we cannot address with the available data. Second, there is the issue of the external validity of our results. We provide here an empirical analysis using recent data on public hospitals operating in the Italian national health care system. Our results are likely to be relevant to public hospitals operating in national health care systems (i.e. massively funded by public revenues), which are prevalent across Europe. However, we are unable to say whether our estimated effects would be confirmed in countries where there is no similar system. Our evaluation analysis could be criticized for not allowing the capture of all the complexities and articulations of the PC model or the specificities of each and every implementation of the general framework of the model. In fact, we claim that our quantitative approach does not substitute but complements more qualitative analyses based, for instance, on ethnographic approaches or case study analyses ([17, 32, 42]). Our approach allows one to gain an assessment of the overall average change of a set of outcomes, controlling for a large range of confounding factors, and to measure the overall effect of the switch to the PC model exploiting the time variation of treated and untreated units and the heterogeneity among MDCs and hospitals.

### Robustness checks

As we mentioned above the adoption of the PC organizational model is not an immediate process but often requires a preparation period as well as a period of adaptation to the new organizational standards. Of the three hospitals that switched all their MDCs to the PC model, two did so in October and one in November 2010. This is the reason why we defined the PC dummy variable for these three hospitals as equal to one for the years 2011 and 2012 only and equal to zero for all the other years. Hence, we tested the robustness of the results by simultaneously dropping both the years 2011 and 2010, which allows for an adjustment period and for a preparation period respectively towards the PC model (Table 6).

**Table 6:** The effect of the PC organizational change, difference-in-difference estimations excluding the years 2010 and 2011

	(1)	(2)	(3)	(4)
Days in hospital	-0.006	0.106***	-0.044***	-0.055***
	[0.028]	[0.008]	[0.010]	[0.012]
No.Obs.	9,434	9,434	9,434	9,434
R-squared	0.945	0.962	0.966	0.967
Re-hospitalization rate (same MDC)	-0.006	0.009***	-0.010***	-0.006**
	[0.004]	[0.002]	[0.002]	[0.003]
No.Obs.	9,434	9,434	9,434	9,434
R-squared	0.943	0.962	0.965	0.967
Re-hospitalization rate (same Hosp., same MDC)	-0.002	0.010***	-0.007***	-0.006***
	[0.004]	[0.001]	[0.002]	[0.002]
No.Obs.	9,434	9,434	9,434	9,434
R-squared	0.878	0.907	0.911	0.916
Mortality rate	0.001	0.008***	0.001	0.001
	[0.002]	[0.001]	[0.002]	[0.002]
No.Obs.	9,434	9,434	9,434	9,434
R-squared	0.887	0.918	0.929	0.932
MDC x Year interaction	No	Yes	Yes	Yes
Hosp. and MDC Interactions with:				
Discharges	No	No	Yes	Yes
Age	No	No	No	Yes
Male	No	No	No	No

*Note:* The table shows the estimate of the coefficient of the PC variable, for different variables, and respectively the log of the number of days of hospitalization (days in hospital), the log of the rate at which a discharged patient happens to be re-hospitalized in the same MDC within 30 days (re-hospitalization rate, same MDC), the log of the rate at which a discharged patient happens to be re-hospitalized in the same hospital and MDC within 30 days (re-hospitalization rate, same Hosp. and same MDC) and the log of the mortality rate within 30 days after discharge (mortality rate). All regressions are estimated always controlling for the year fixed effects, average number of discharged patients, average age and share of male patients for MDC *j*, hospital *h* and year *t*. Additional interaction terms have been added sequentially, as shown at the bottom of the table. Standard errors have been clustered at the hospital level

* 10% significance level, ** 5%, *** 1%

The results show that the main findings for both efficiency and effectiveness of the PC model are broadly confirmed, showing only a slightly larger effect of the PC innovation on the average length of hospital stay. Also results on effectiveness show the overall robustness of results to the exclusion of the years 2010–2011 (Table 6). Finally, observing that our sample size is affected by the fact that many MDC-year cells present fewer than 30 HDCs per year and that small denominators (MDCs with very few patients in any one year) may introduce statistical noise into our outcome indicators - and for these reasons have been dropped from the analysis - we estimate the same empirical models allowing for different minimum cell sizes. The results are presented in Tables 7 and 8 and again produce evidence of overall strong robustness of our estimates.

**Table 7:** The effect of the PC organizational change, difference-in-difference estimations selecting different minimum cell sizes

	(1)	(2)
	Min cell size = 20	Min cell size = 40
Days in hospital	-0.036***	-0.082***
	[0.009]	[0.011]
No.Obs.	12,707	11,175
R-squared	0.961	0.971
Re-hospitalization rate (same MDC)	-0.007***	-0.009***
	[0.002]	[0.002]
No.Obs.	12,707	11,175
R-squared	0.961	0.971
Re-hospitalization rate (same Hosp., same MDC)	-0.008***	-0.007***
	[0.002]	[0.002]
No.Obs.	12,707	11,175
R-squared	0.899	0.921
Mortality rate	-0.000	-0.004*
	[0.002]	[0.002]
No.Obs.	12,707	11,175
R-squared	0.916	0.939
MDC x Year interaction	Yes	Yes
Hosp. and MDC Interactions with:		
Discharges	Yes	Yes
Age	Yes	Yes
Male	Yes	Yes

*Note:* The table shows the estimate of the coefficient of the PC variable, for different variables, and respectively the log of the number of days of hospitalization (days in hospital), the log of the rate at which a discharged patient happens to be re-hospitalized in the same MDC within 30 days (re-hospitalization rate, same MDC), the log of the rate at which a discharged patient happens to be re-hospitalized in the same hospital and MDC within 30 days (re-hospitalization rate, same Hosp. and same MDC) and the log of the mortality rate within 30 days after discharge (mortality rate). All regressions are estimated always controlling for the year fixed effects, average number of discharged patients, average age and share of male patients for MDC *j*, hospital *h* and year *t*. Additional interaction terms have been added sequentially, as shown at the bottom of the table. Standard errors have been clustered at the hospital level

* 10% significance level, ** 5%, *** 1%

**Table 8:** The effect of the PC organizational change, difference-in-difference estimations selecting different minimum cell sizes excluding the years 2010 and 2011

	(1)	(2)
	Min cell size = 20	Min cell size = 40
Days in hospital	-0.043***	-0.085***
	[0.011]	[0.013]
No.Obs.	9,900	8,695
R-squared	0.962	0.972
Re-hospitalization rate (same MDC)	-0.009***	-0.009***
	[0.002]	[0.003]
No.Obs.	9,900	8,695
R-squared	0.962	0.972
Re-hospitalization rate (same Hosp., same MDC)	-0.009***	-0.005***
	[0.002]	[0.002]
No.Obs.	9,900	8,695
R-squared	0.907	0.927
Mortality rate	-0.001	-0.002
	[0.002]	[0.002]
No.Obs.	9,900	8,695
R-squared	0.922	0.942
MDC x Year interaction	Yes	Yes
Hosp. and MDC Interactions with:		
Discharges	Yes	Yes
Age	Yes	Yes
Male	Yes	Yes

*Note:* The table shows the estimate of the coefficient of the PC variable, for different variables, and respectively the log of the number of days of hospitalization (days in hospital), the log of the rate at which a discharged patient happens to be re-hospitalized in the same MDC within 30 days (re-hospitalization rate, same MDC), the log of the rate at which a discharged patient happens to be re-hospitalized in the same hospital and MDC within 30 days (re-hospitalization rate, same Hosp. and same MDC) and the log of the mortality rate within 30 days after discharge (mortality rate). All regressions are estimated always controlling for the year fixed effects, average number of discharged patients, average age and share of male patients for MDC *j*, hospital *h* and year *t*. Additional interaction terms have been added sequentially, as shown at the bottom of the table. Standard errors have been clustered at the hospital level

* 10% significance level, ** 5%, *** 1%

One can notice that the effects on re-hospitalization rates (both the same MDC and the same hospital-MDC) are largely unaffected by the different cell sizes. The signs do not change and the statistical significance of these indicators is roughly constant, between 20 and 40 minimum cell sizes, and equal to the baseline selection of Table 5. As for the size of the reduction in mortality and the length of the hospital stay, it is positively correlated with the cell size, suggesting that the higher the restriction, the stronger and more significant is the estimated effect, implying that the adoption of a PC organizational model has stronger effects in relatively larger MDCs.

## Discussion

Patient-Centered care has been widely embraced by many of the industry’s most influential care providers, policymakers, regulatory agencies, research bodies, and funders. This profound shift can be traced to a 2001 Institute of Medicine report ([43]) that identified a focus on Patient-Centered care as one factor constituting high-quality care. This solidified the Patient-Centered care approach not only as a way of creating a more appealing patient experience, but also as a fundamental practice for the provision of high-quality care, with direct implication on hospital organizational models and processes ([44]). In this paper we took advantage of the fortunate coincidence of a quasi-experimental setting regarding all the MDCs in three hospitals of an important region of Italy and of the availability of a unique administrative data set to develop an ex post evaluation of an innovation from a traditional functional model to a PC organizational model in hospitals. We suggested a quantitative framework for overcoming some of the current challenges in the evaluative policies of hospital organizational models (for a similar approach to policy analysis in health care see [45]). To the best of our knowledge, this is the first quantitative assessment of such an important and frequently found organizational setting in hospitals.

We managed to estimate difference-in-difference models that support some of the theoretical claims of the PC model as a whole. In particular, the PC model seems to have an effect on effectiveness, which is a relevant dimension of the quality of health care services. The rate of readmission for PC hospitals decreases slightly, by less than 1%, with no significant effect on the death rate of patients. The strongest effects are found in the efficiency variable measuring the duration of hospitalization. These results are in line with the theoretical framework outlined in the Empirical Model subsection, which suggested increased efficiency and effectiveness of PC hospitals. In particular, the increase in efficiency emerges from the reduction of the hospitalization duration. As for efficacy, our results, showing a reduction in re-hospitalization, suggest an increased level of efficacy of hospitals that switched to a PC organization. The lack of statistical significance of mortality rates suggests that this organizational innovation is unlikely to have any impact on such an outcome.

Considering PC model change as a relevant turning point with respect to hospital managing and policy making, and considering also the extension of its implementation and debate in European countries and international context (as we have seen, experiments can be found in England ([22]), in the Netherlands ([23]), in Spain ([24]), in Sweden ([25]) and in Italy), we advocate the relevance of this paper’s attempt in two directions. First, this paper fills the quantitative assessment gap related to the PC hospital model with a specific focus on efficiency and effectiveness. Such an organizational change towards the PC model can be a costly process, implying a rebalancing of responsibilities and power among hospital personnel, affecting inter-disciplinary and inter-professional relations (e.g. medical and nursing staff) and possibly affecting individual motivations and enthusiasm or opposition to the change ([28]). Nevertheless, our results confirm the effect of these hospital innovations on efficiency ([11]), adding some robust results, thus suggesting that a change to the PC model can be worthwhile. This evidence can be used to inform and sustain hospital managers and policy makers in their hospital design efforts, and to communicate the innovation advantages within the hospital organizations, among the personnel and in the public debate. With these data analysis, we believe that this health care innovation can be regarded as an actual improvement to meet the needs of the community, contrasting the possible perception that it may have been driven by managerial, international or political trends. As suggested by McKee and Healy ([36]), all that we can be certain of is that the hospital of the future will be different from the hospital of today and the PC model is an interesting innovation, which, however, requires a proper evaluation.

Second, this research exercise can be also considered as a guiding example for ex-post evaluation of broad interventions. This is a complicated task, although worthwhile as it provides fundamental suggestions to policy makers engaged in important future and complex innovations ([46]). This study refers to the long-standing tradition of program evaluation, which may be used when the real-world provides data to support testing hypothesis with a counterfactual approach. The availability of administrative data, which is increasing in all developed countries and is characterised by little measurement error and high detail of information, makes the opportunity for sound quantitative assessments, offering evidence that turns useful in the planning of innovation initiatives and their policy implications for the overall society.

## Conclusions

This paper provides a quantitative estimation of efficiency and effectiveness changes following the implementation of the PC hospital model in a major region of Italy. Taking advantage of a quasi-experimental setting and a detailed administrative dataset, we perform an ex-post evaluation of innovating the hospital organization by switching from a traditional functional model to a PC organizational one. We provide robust evidence, at the average MDC, of a statistically significant and positive effect of the introduction of the PC model on both effectiveness and efficiency. In particular, the increase in efficiency emerges from the reduction of the average length of stay, while for efficacy, our results, show a reduction in re-hospitalization rates of hospitals that switched to a PC organization. These results are in line with our theoretical framework which suggests an increase in efficiency and effectiveness of PC hospitals and provides a sound example of a quantitative evaluation of an organizational intervention adopting a counterfactual approach.

## Abbreviations

PC: Patient-centered
MDC: Major diagnostic categories
HDC: Hospital discharge charts
DRG: Diagnosis-related group

## Footnotes

1 MDC codes are internationally recognized thanks to their adoption in the United States medical care reimbursement system. They are formed mapping all the DRG codes into 25 mutually exclusive diagnosis areas.

2 We estimate log-linear models of the outcome means considering that the outcomes that we use are strictly non-negative (e.g. means of count variables or rates), not over-dispersed and do not raise zero inflation concerns ([47], p. 645)

3 The coefficient of interest, *γ*, refers to a dummy variable, *P**C*_*h*,*t*_, that is equal to one for those hospitals that adopted a PC model in the years immediately after their organizational change and zero otherwise. This is clearly equivalent to including a standard interaction term between the treatment variable and a post-reform dummy. Also notice that there is no need to include a treatment dummy, as we have the full set of hospital fixed effects, or a post-reform dummy variable, as we have the full set of year fixed effects

4 Data are provided by the Health Care Department of the Lombardy Region and are processed in collaboration with CRISP - the Inter-university Research Centre on Public Services at the University of Milan-Bicocca (Italy). Individual HDC records are not publicly available under the Italian privacy law. The Health Care Department of the Lombardy Region must be contacted to discuss the provision of the data

5 The diagnosis-related group (DRG) code is a standard classification ([48]) adopted in the Lombardy Region of Italy since 1995. The DRG classifies hospital discharge charts depending on patients’ diagnoses, procedures, complications, co-morbidity and demographic factors (such as age and gender)

6 In fact, HDC data trace the department that is in charge of each patient and record the total number of departmental transfers of each HDC, but not whether a transfer is in fact a bed change within the same hospital or, more simply, a change of the administratively responsible department.

7 An important efficiency measure that we do not observe is the cost of single HDCs as we have no information on the composition and cost of the physical and human resources used. In fact, we are provided with the cost of reimbursement by the Lombardy Health Care System to hospitals for each HDC, but this variable is unsuitable for use as a cost measure as it is affected by DRG up-coding practices, discretionality of the regional policy makers in deciding the price of the duration and the DRG of each HDC, allowing for strategic behaviour of hospital managers. For an extensive analysis of the reimbursement mechanism adopted in the Lombardy Health Care System, see [49]

8 The attractiveness of the Lombardy Health Care System is indeed relevant, with a proportion of hospitalized patients from other regions close to 10% ([49]) of the yearly provision. The main reason for dropping the HDCs of patients with residence outside Lombardy is because they might be occasional users of the Lombardy Health Care System and we lack relevant information about them regarding their possible re-hospitalization and death. For instance, as we know the date of death of Lombardy residents only, including non-Lombardy patients would bias the average mortality rate of patients downward by an unpredictable amount. We also dropped one-day-long and subacute HDCs due to comparability issues.

9 A similar approach was used by [50]

10 Some robustness checks assessing the relevance of this selection rule are provided in Tables 7 and 8.

11 We developed this test (results in Table 4) for all the models that we estimated in Table 5 (columns 1 to 4), starting from the basic equation (Eq. 1) to the saturated equation (Eq. 2), as follows. First, we computed each outcome variable of interest after partialling out the contribution of all the independent variables except for *P**C*_*h*,*t*_. Hence, we regressed each of them on a fourth-degree polynomial time trend, allowing all the coefficients to differ between the PC and the traditionally organized hospitals (unrestricted model), and we regressed the same dependent variable on a fourth-degree polynomial time trend in which only the intercept is allowed to differ between the two groups considered. Finally, we computed the statistic $\left (\left (\left (R^{2}_{UR}-R^{2}_{R}\right)/r\right)/\left (1-R^{2}_{UR}\right)\right)$, which is distributed as an F-distribution with (*r*,*n*−*k*) degrees of freedom and in which $R^{2}_{R}$ and $R^{2}_{UR}$ are respectively the *R*^2^ of the restricted and unrestricted models, *r* is the number of restrictions imposed and *n*−*k* is the number of degrees of freedom of the unrestricted model.
